# Acquired Immunodeficiency Syndrome (AIDS)-Related Kaposi’s Sarcoma in Conjunction With Methicillin-Resistant Staphylococcus aureus (MRSA) and May-Thurner Syndrome

**DOI:** 10.7759/cureus.60114

**Published:** 2024-05-11

**Authors:** Tristan B Minick, Robert A Norman

**Affiliations:** 1 Dermatology, University of Florida, Gainesville, USA; 2 Dermatology, Nova Southeastern University Dr. Kiran C. Patel College of Osteopathic Medicine, Fort Lauderdale, USA

**Keywords:** may-thurner syndrome, pvd: peripheral vascular disease, hiv aids, anemia, kaposi sarcoma management

## Abstract

A 34-year-old male with a history of peripheral vascular disease and multifactorial anemia presented with red blotches on his face, trunk, and extremities, multiple large bumps prominent on the lower extremities that burst at times with yellow pus and blood, swelling in the ankles, extremely dry feet, a chronic ulcer on the foot, and a dry, flaky, and irritated left middle finger. The patient was human immunodeficiency virus (HIV) positive, viral load undetectable. Endovenous laser ablation therapy was performed to correct venous insufficiency. A balloon was placed in the common iliac vein to treat May-Thurner syndrome. The bumps on the lower extremities were biopsied and found to be Kaposi's sarcoma (KS) and were removed by both wide excisions and shave removals, and further treatment with doxorubicin was performed successfully. The foot ulcer was found to be positive for methicillin-resistant *Staphylococcus aureus* (MRSA) and was treated with sulfamethoxazole-trimethoprim, metronidazole, and a chlorhexidine topical liquid. The patient noted that the treatments on his leg were working very well, and he was clearing up.

## Introduction

May-Thurner syndrome is a condition that is generally caused by the right iliac artery or vertebral body compressing the left iliac vein. This can often cause venous valve insufficiency where blood pools in the legs and varicose veins in the lower extremities. Some of the most common clinical symptoms are deep vein thrombosis, edema, pain, varicose veins, and ulcers in the left lower limb [1]. Fibrous changes in the venous wall structure can also sometimes be observed [2]. Common treatments for the condition include catheter-directed thrombolysis and placement of stents supplemented by long-term anticoagulation therapy and graduated compression stockings [1].

Kaposi's sarcoma (KS) is a multifocal vascular tumor involving the skin and other organs, and it is the most common neoplasm in patients with acquired immunodeficiency syndrome (AIDS) [3]. It is an endothelial cell cancer with an inflammatory component and highly heterogeneous histopathology and clinical behavior. There are four main epidemiological forms of KS that are now widely recognized: AIDS-related/epidemic/human immunodeficiency virus (HIV)-associated, iatrogenic, endemic, and classic/sporadic [4]. In this report, we will focus on the first variant. HIV-associated KS is considered to be one of the AIDS-defining conditions [3]. Widespread use of antiretrovirals to control HIV reduced the prevalence of AIDS-related KS, though KS does still occur in patients with well-controlled HIV infections. The etiologic agent, or the agent that causes KS, is the KS herpesvirus (KSHV, also known as human herpesvirus-8 (HHV-8)). Viral changes can induce KS-associated cellular changes that enable the virus to evade the host immune system and allow the infected cell to survive and proliferate despite viral infection. In advanced-stage KS, chemotherapy with pegylated liposomal doxorubicin or paclitaxel is the most common treatment, though it is rarely curative. The classic clinical presentation for AIDS-related KS is multiple cutaneous lesions on the limbs, trunk, and face. It is also common to see mucosal lesions and visceral involvement, or the involvement of the soft tissues of the body. Tumor-associated edema is another possible symptom. The risk of developing KS increases as CD4 cell count declines but decreases with the use of combination antiretroviral therapy (cART). The disease may follow an indolent course but visceral involvement is not uncommon and may be aggressive. The KS may regress with effective cART treatment [4]. In this case, the patient had dozens of KS lesions in a setting of elephantiasis-like changes in the lower extremities. When there are widespread KS lesions on the skin, intravenous chemotherapy may be indicated [5].

*Staphylococcus aureus* is a major human pathogen that can acquire resistance to most antibiotics and is a gram-positive, coagulase-positive bacteria. The clinical use of methicillin has led to the appearance of methicillin-resistant *Staphylococcus aureus* (MRSA). Over the past few decades, this bacterium has created many new clones that have gained the ability to infect people without predisposing risk factors in areas outside of clinical settings. Infections that arise from MRSA are associated with higher mortality rates and longer hospital stays than other methicillin-susceptible strains. The methicillin resistance stems from the production of an altered penicillin-binding protein (PBP) that is associated with decreased affinity for most semisynthetic penicillins. The added resistance to the bacteria has posed a challenge for the treatment of its infections due to the resistance against all available penicillins and most beta-lactam drugs other than ceftaroline and ceftobiprole. Patients infected with the less resistant community-associated MRSA (CA-MRSA) can be treated with aminoglycosides, erythromycin, clindamycin, and fluoroquinolones. Patients with the more serious healthcare-associated MRSA (HA-MRSA), however, have fewer options and have historically been treated with vancomycin [6].

## Case presentation

A 34-year-old male presented with venous insufficiency that had a gradual onset over the course of a few years. The symptoms included aching and swelling in both lower extremities, which got worse towards the end of the day and with walking. This caused a change in skin color, itching, and swelling. The symptoms were relieved by rest and elevation. The patient also had varicose veins in both lower extremities. The patient received endovenous laser ablation therapy (EVLT) on the left and right greater saphenous veins, which were successfully closed. There was no evidence of color flow seen during a follow-up venous lower extremity deep venous thrombosis (DVT) study in combination with the use of 20/30 compression stockings.

Another DVT study performed six weeks later revealed occlusive, acute DVT in the left posterior tibial veins, occlusive, acute superficial vein thrombus in a mid and distal medial thigh varicosity, a mid-medial calf varicosity, and a mid-thigh perforating vein, and large, non-vascularized lymph nodes in the left groin and proximal thigh. The patient indicated that he had been experiencing shortness of breath for over 24 hours and swelling and pain in his left leg for around 48 hours. The patient had multiple axial computed tomography (CT) images taken of his abdomen and pelvis without intravenous (IV) contrast along with axial thin slice images with IV contrast and CT angiography of the abdomen, pelvis, and bilateral lower extremities. This imaging found that the bilateral great saphenous veins were enlarged with no evidence of arteriovenous (AV) fistula and some hypertrophy of the adrenal glands with a possible mass in the left gland. There were also multiple prominent retroperitoneal lymph nodes measuring up to 8 mm and multiple bilateral iliac and inguinal lymph nodes that were enlarged. Notably, there was a left common iliac node measuring 1.5 cm, a right common iliac node measuring 1.3 cm, and a large left inguinal node measuring 1.9 cm. The 3D maximum intensity projection (MIP) images that were taken confirmed these findings.

A diagnosis of May-Thurner syndrome was made following the results of inferior vena cava (IVC) ultrasounds that found that the left common iliac vein was reduced in diameter compared to the contralateral side, suggesting possible iliac vein compression. An intravascular ultrasound catheter was used to examine the patient's veins from the IVC and an area of mild compression in the common iliac vein with a 12% stenosis was found. The area of compression was then ballooned with a 14x4 balloon to minimal atmospheres.

**Figure 1:**
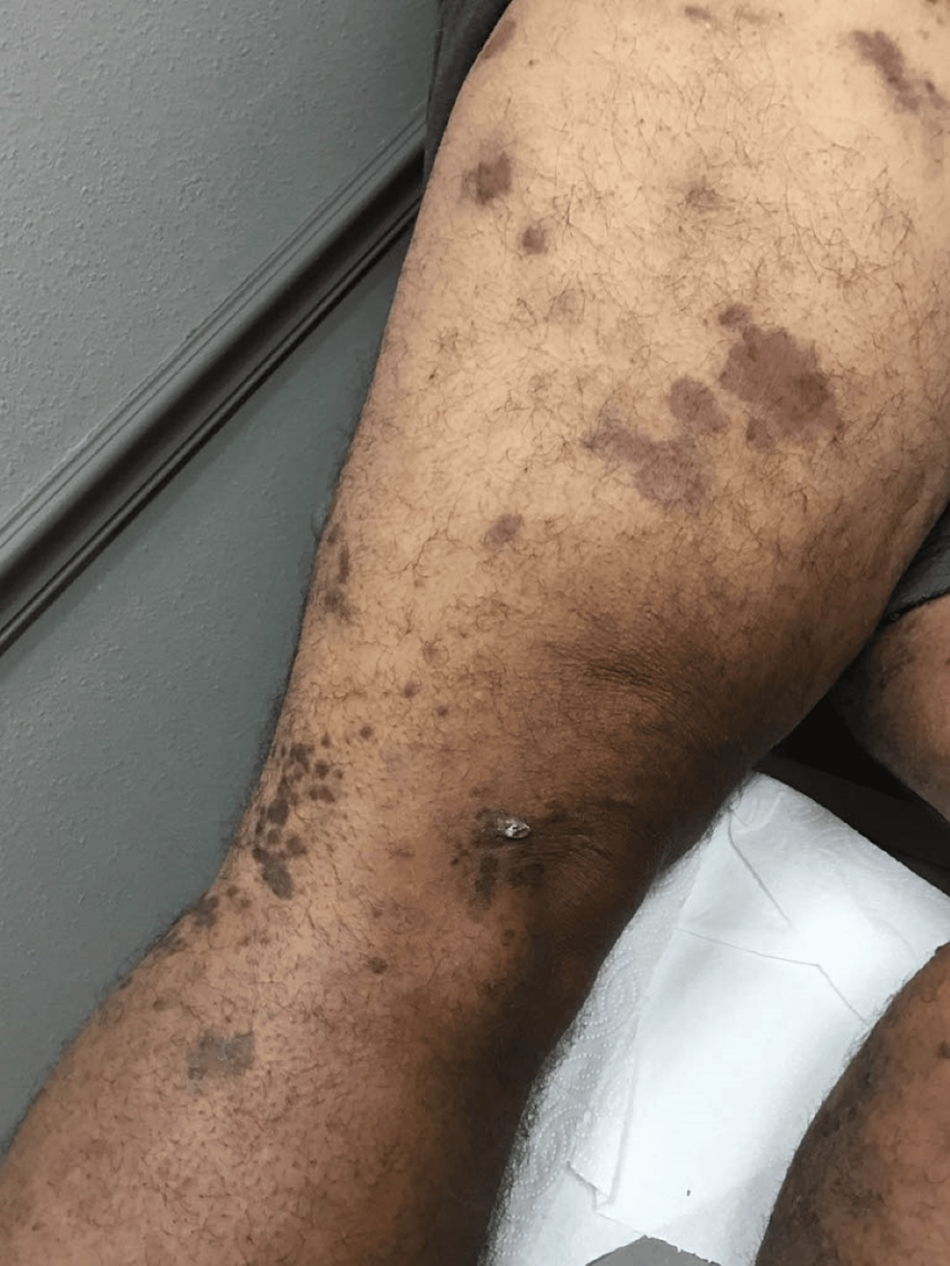
Red blotches on the right leg

**Figure 2:**
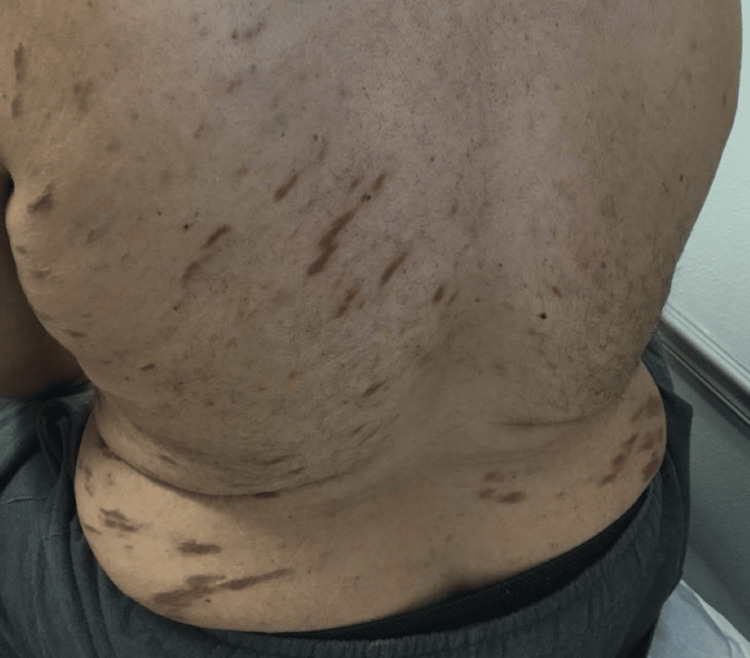
Red blotches on the back

**Figure 3:**
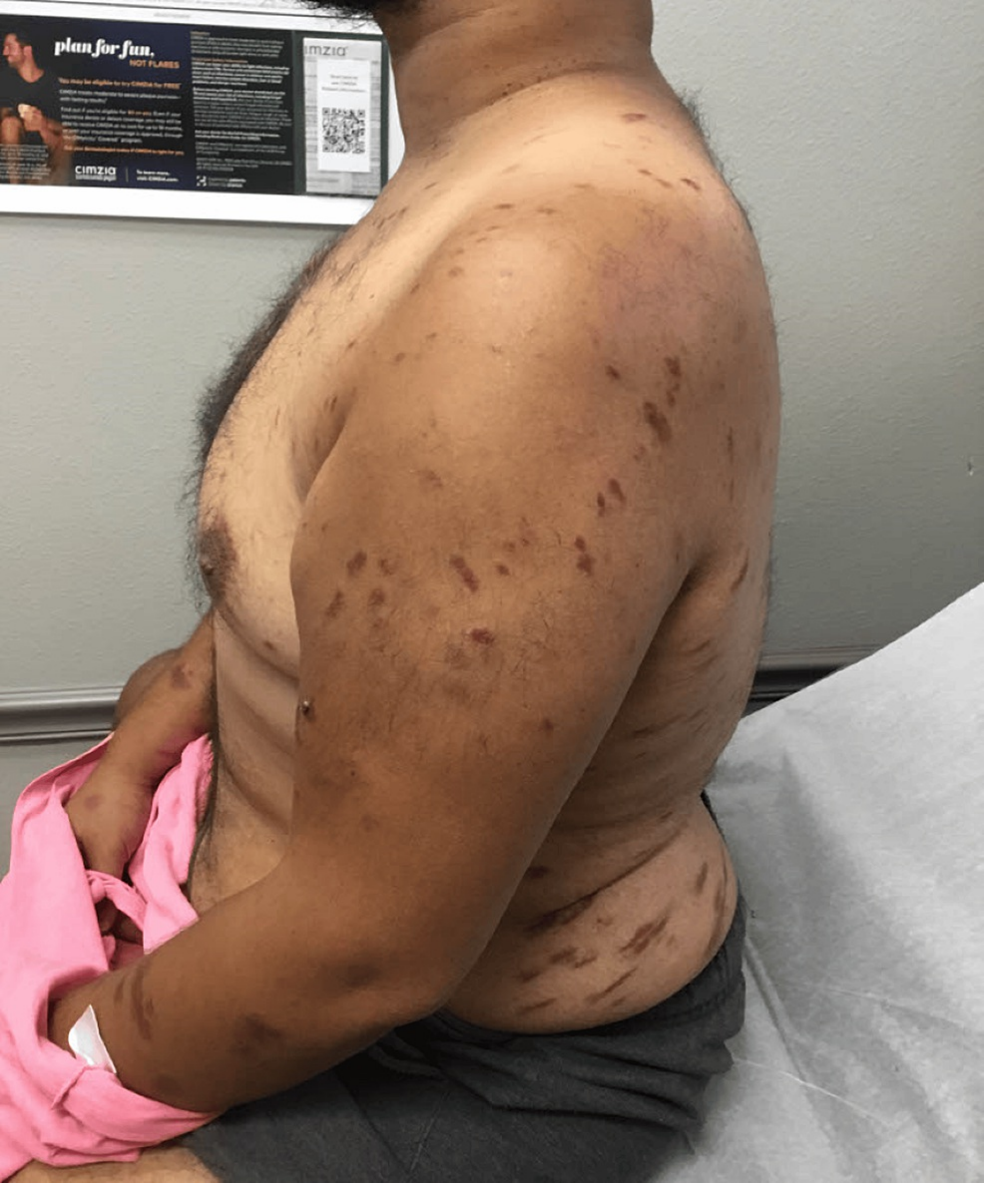
Red blotches on the left arm

**Figure 4:**
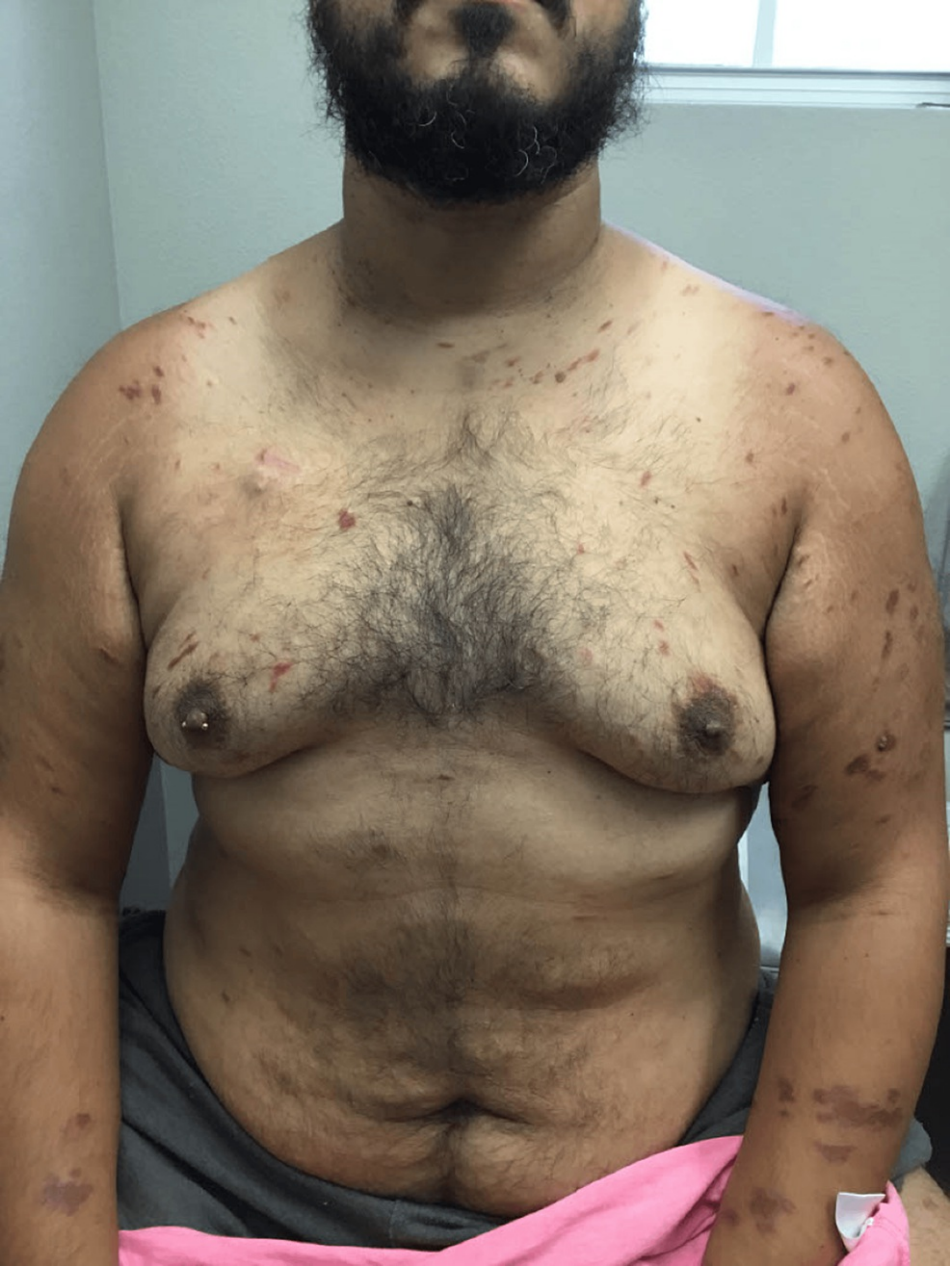
Red blotches on the chest

The patient presented to the dermatologist with red blotches on his face, trunk, and extremities, multiple large bumps prominent on the lower extremities that burst at times with yellow pus and blood, swelling in the ankles, extremely dry feet, and a dry, flaky, and irritated left middle finger (Figures 1-4). His current condition first appeared 1.5 years prior to the visit after a COVID-19 infection. The patient was HIV positive, undetectable. On physical examination, it was noted that the patient had red and inflamed lower extremities with itching and tenderness, and there was abnormally dry skin present on all four extremities with localized skin redness and discoloration on the abdomen.

Ammonium lactate 12% lotion was prescribed for daily use on the areas of skin with excessive dryness. Tangential shave biopsies were taken on the large, inflamed growths present on the patient's left lower leg and left inner foot. The sample from the lower leg was consistent with the top of the intravascular papillary endothelial hyperplasia of Masson, and the sample from the inner foot was consistent with the top of an inflamed angioma. Triamcinolone acetonide 0.1% topical ointment was prescribed for the intravascular papillary endothelial hyperplasia on the bilateral legs. Wide excisions were performed on the patient's left lower leg and revealed findings consistent with KS. Mupirocin 2% topical ointment and cefalexin 500 mg oral capsules were prescribed to prevent infection. Shave removals were completed on two additional KS on the patient's left medial foot and left lateral foot. A positron emission tomography/computed tomography (PET/CT) scan showed bilateral cervical axillary, pelvic, and inguinal enlarged lymph nodes with hypermetabolic activity suggestive of metastatic disease. There was also thickening of the left adrenal gland with hypermetabolic uptake, hypermetabolic activity of the nodular thickening of the lower extremity's skin, and the subcutaneous tissue on the left was greater than the right.

**Figure 5:**
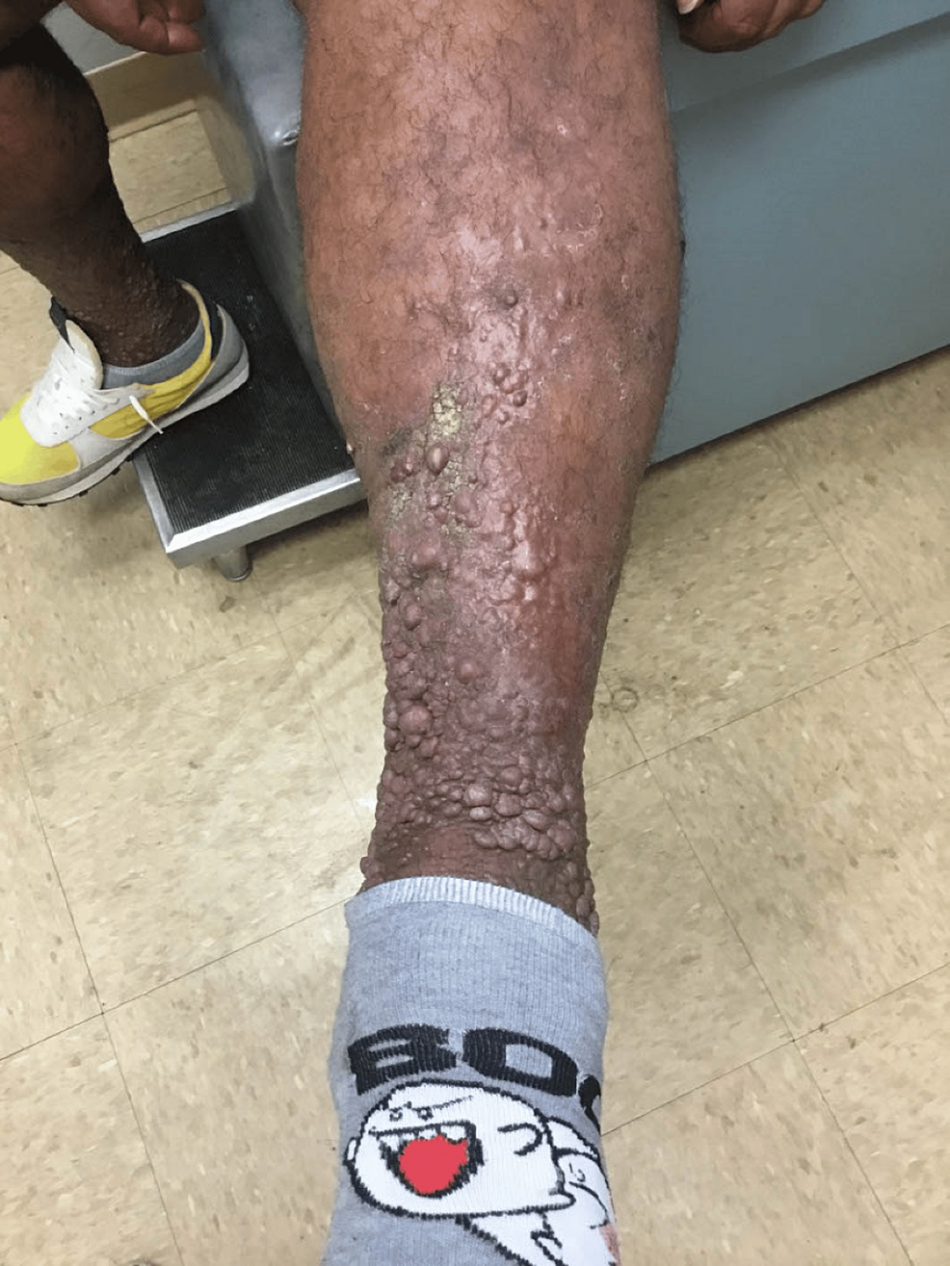
Ulcers on the left leg

**Figure 6:**
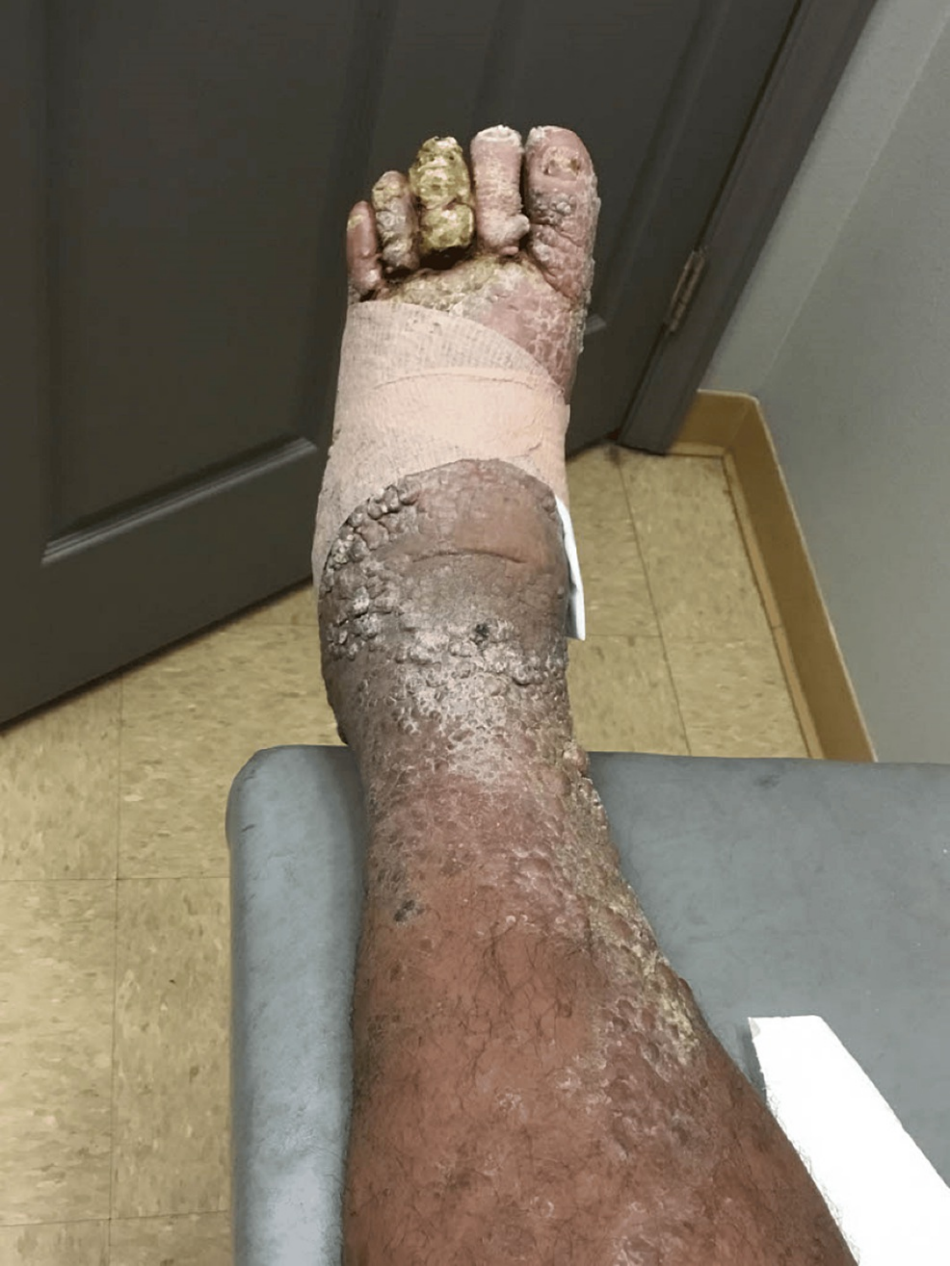
Top of the left foot

**Figure 7:**
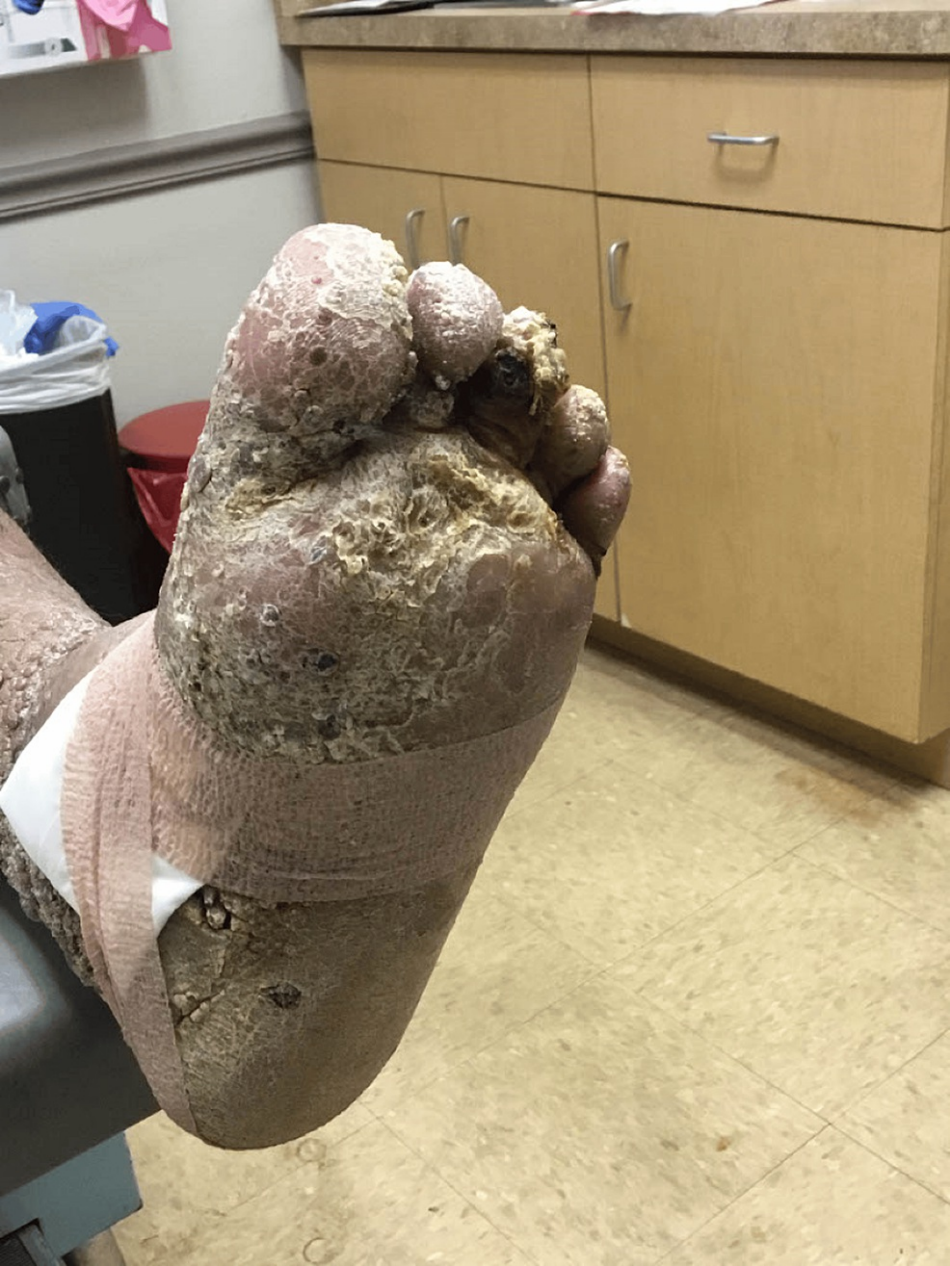
Bottom of the left foot

This combination of findings was consistent with a diagnosis of KS with nodal involvement, and the patient underwent therapy with single-agent doxorubicin which was well-tolerated, and improvement of the disease was noted within a week of the first treatment. The patient also presented with chronic foot ulcers that were causing significant pain (Figures 5-7). Chlorhexidine 4% topical liquid and metronidazole 0.75% topical gel were prescribed, and a culture was performed on the ulcer that revealed a positive result for MRSA. On follow-up, less odor was noted, and the ulcers appeared to have healed significantly. A sulfamethoxazole-trimethoprim tablet was prescribed to help treat the infection following the results of a culture and sensitivity test (Table 1).

**Table 1:** MRSA culture and sensitivity test MRSA: Methicillin-resistant *Staphylococcus aureus*

MRSA Culture and Sensitivity	
	Minimum Inhibitory Concentration
Ciprofloxacin	1
Clindamycin	≤0.25
Erythromycin	≥8
Gentamicin	≤0.5
Tetracycline	≤1
Trimethoprim/Sulfa	≤10
Vancomycin	≤0.5

Six weeks following the sixth and final cycle of the doxorubicin-based therapy, the patient returned for a follow-up visit and noted no progression or new lesions. The patient also indicated that he had noticed further improvement in his previously noted lesions in the two to three weeks prior to the visit. A 2D echocardiogram was obtained that revealed a normal though declining ejection fraction at 55-60% compared to the 60-65% that was observed six months prior, just before beginning the doxorubicin-based therapy.

## Discussion

Here, we present a 34-year-old male who is HIV positive, undetectable with May-Thurner syndrome, extensive KS, and an MRSA infection. The May-Thurner syndrome was successfully treated by ballooning the area of the common iliac vein that was compressed with a 4x14 balloon. There were too many KS for wide excisions, so the patient was treated with doxorubicin. The MRSA infection was treated with a combination of a chlorhexidine topical liquid and metronidazole topical gel. This combination of multiple major conditions all occurring at once is highly unusual and is the point of interest for this case.

May-Thurner syndrome, the compression of the left iliofemoral vein by the right common iliac artery, is an uncommon disorder that frequently goes unnoticed. It is estimated to cause 2-5% of all DVT based on the times that it is caught, but retrospective studies have estimated the actual prevalence to be 14-32%. It is also reported to be almost twice as common in women as it is in men [7]. There is still much to be learned about the signs of May-Thurner syndrome and how it can be treated, and it was fortunate to have it diagnosed using IVC ultrasounds while the patient was displaying symptoms from multiple conditions at once.

MRSA is a bacterium that can be very difficult to treat due to its resistance to a range of common antibiotics. Vancomycin is typically the drug of choice to treat this infection [8], though other options include daptomycin, ceftaroline, linezolid, telavancin, fosfomycin, and quinupristin-dalfopristin [9]. In this case, the patient was treated with a combination of chlorhexidine topical liquid, metronidazole, and sulfamethoxazole-trimethoprim, which helped to eliminate the very disturbing odor of the infection that the patient complained about. Given the abnormally large number of sarcomatous lesions and his history of May-Thurner syndrome, it is possible that this underlying pathology contributed to the widespread nature of his infections. This aspect of the case should be investigated further in similar patients to determine if there is any link between these conditions and if more preventative measures or increased screening could be used when treating patients with similar presentations. This could potentially allow for earlier diagnosis and treatment of KS or May-Thurner syndrome to limit the negative impact on the patients.

## Conclusions

Here, we presented a rare case of a patient who had many KS, May-Thurner syndrome, and an MRSA infection all at the same time. This case demonstrates the importance of coordination between multiple disciplines in the diagnosis and treatment of complicated cases such as this. It was vital that each of the many simultaneous symptoms were considered separately to discover the three separate diagnoses for this patient.

More research should be done to consider the possible linked nature of these three conditions that may have led to such an unusual presentation. This case could possibly be indicative that the conjunction of May-Thurner syndrome or MRSA with KS led to an abnormally large number of lesions present on this patient at once and could lead to potential new preventative or diagnostic measures in similar patients in the future.
